# Pelanserin: 3-[3-(4-phenyl­piperazin-1-yl)prop­yl]quinazoline-2,4(1*H*,3*H*)-dione

**DOI:** 10.1107/S160053681401602X

**Published:** 2014-07-23

**Authors:** Gerardo Aguirre Hernández, Ratnasamy Somanathan, Sylvain Bernès

**Affiliations:** a Centro de Graduados e Investigación del Instituto Tecnológico de Tijuana, Apdo. Postal 1166, 22500 Tijuana, B.C., Mexico; b DEP Facultad de Ciencias Químicas, UANL, Guerrero y Progreso S/N, Col. Treviño, 64570 Monterrey, N.L., Mexico

**Keywords:** crystal structure

## Abstract

The title compound, C_21_H_24_N_4_O_2_, is a potent serotonin 5-HT_2_ and α_1_-adrenoceptor antagonist. The *n*-propyl chain links the quinazolinedione heterocycle and the phenyl­piperazine group in which the benzene ring is equatorially located and the piperazine ring has the expected chair conformation. The dihedral angle between the planes of the benzene ring and the quinazolinedione ring system is 74.1 (1)°. In the crystal, mol­ecules form centrosymmetric dimers through *R*_2_^2^(8) hydrogen-bonded rings involving the amine and one carbonyl group of the quinazolinedione moiety. These dimers are extended into chains extending along the *a*-axis direction through expanded centrosymmetric cyclic C—H⋯O associations involving the second carbonyl group, giving *R*_2_^2^(20) and *R*_1_^2^(7) motifs.

## Related literature   

For the synthesis of pelanserin, see: Cortez *et al.* (1991 ▶); Garcia *et al.* (2000 ▶); Li *et al.* (2011 ▶). For the pharmacology of pelanserin, see: Flores-Murrieta *et al.* (1990 ▶, 1992 ▶); Villalobos-Molina *et al.* (1995 ▶). For the structure of quinazoline-2,4(1*H*,3*H*)-dione, see: Liu (2008 ▶).
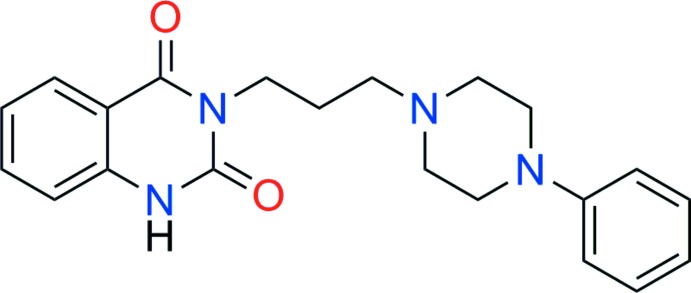


## Experimental   

### 

#### Crystal data  

C_21_H_24_N_4_O_2_
*M* *_r_* = 364.44
Monoclinic, 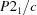

*a* = 15.7531 (17) Å
*b* = 5.4345 (10) Å
*c* = 22.756 (3) Å
β = 104.506 (9)°
*V* = 1886.0 (5) Å^3^
*Z* = 4
Mo *K*α radiation
μ = 0.09 mm^−1^
*T* = 296 K
0.60 × 0.30 × 0.10 mm

#### Data collection  

Bruker P4 diffractometer
3452 measured reflections
3323 independent reflections
1301 reflections with *I* > 2σ(*I*)
*R*_int_ = 0.077
3 standard reflections every 97 reflections intensity decay: 1%

#### Refinement  

*R*[*F* ^2^ > 2σ(*F* ^2^)] = 0.072
*wR*(*F* ^2^) = 0.148
*S* = 0.99
3323 reflections
247 parameters
H atoms treated by a mixture of independent and constrained refinement
Δρ_max_ = 0.18 e Å^−3^
Δρ_min_ = −0.19 e Å^−3^

### 

Data collection: *XSCANS* (Siemens, 1996 ▶); cell refinement: *XSCANS*; data reduction: *XSCANS*; program(s) used to solve structure: *SHELXS2013* (Sheldrick, 2008 ▶); program(s) used to refine structure: *SHELXL2013* (Sheldrick, 2008 ▶); molecular graphics: *Mercury* (Macrae *et al.*, 2008 ▶); software used to prepare material for publication: *SHELXL2013*.

## Supplementary Material

Crystal structure: contains datablock(s) I, global. DOI: 10.1107/S160053681401602X/zs2304sup1.cif

Structure factors: contains datablock(s) I. DOI: 10.1107/S160053681401602X/zs2304Isup2.hkl

Supporting information file. DOI: 10.1107/S160053681401602X/zs2304Isup3.cml

CCDC reference: 1013055

Additional supporting information:  crystallographic information; 3D view; checkCIF report

## Figures and Tables

**Table 1:** Hydrogen-bond geometry (Å, °)

*D*—H⋯*A*	*D*—H	H⋯*A*	*D*⋯*A*	*D*—H⋯*A*
N3—H3⋯O2^i^	0.95 (4)	1.85 (4)	2.799 (5)	171 (4)
C18—H18*A*⋯O10^ii^	0.97	2.71	3.625 (6)	157
C25—H25*A*⋯O10^ii^	0.93	2.59	3.404 (6)	147

Symmetry codes: (i) 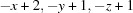; (ii) 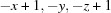.

**Fig. 1.**
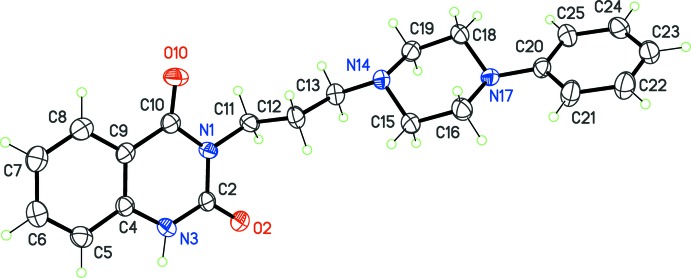
Molecular structure of the title compound, with 30% probability level displacement ellipsoids for non-H atoms.

**Fig. 2.**
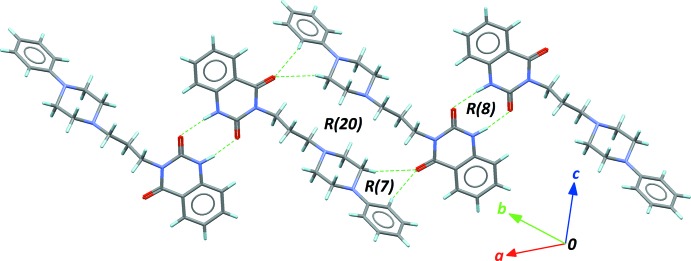
Part of the crystal structure, showing hydrogen bonds as dashed lines.

## supplementary crystallographic information

### S1. Comment

Quinazolinediones are important heterocycles, which have been shown to possess pharmacologically interesting properties, displaying for example anti-hypertensive, or antidiabetic activity. Among these, synthetic pelanserin (TR-2515) is a well-established potent antihypertensive agent, a feature attributed to its 5-HT_2_ and α_1_-adrenoceptor antagonist activity (Flores-Murrieta *et al.*, 1990, 1992; Villalobos-Molina *et al.*, 1995). Indeed, this molecule presents an activity comparable to that of ketanserin, a clinically used drug. Both molecules also share the quinazoline-2,4-dione scaffold.

We synthesized pelanserin *via* a ring closure procedure we have developed, based on the reaction between an *o*-aminobenzamide and triphosgene (Cortez *et al.*, 1991; Garcia *et al.*, 2000). Such a strategy has also been used starting from isatoic anhydride and a readily available primary amine, with triphosgene as ring closure agent (Li *et al.*, 2011).

The title compound has the expected conformation, with the extended *n*-propyl chain linking the heterocyclic systems (Fig. 1). The quinazolinedione group has the same geometry as that observed for free quinazoline-2,4(1*H*,3*H*)-dione (Liu, 2008), and the piperazine ring is found in the chair conformation, with the phenyl substituent group equatorially located. Both lone pairs in the piperazine ring are thus placed in axial positions. The dihedral angle between phenyl and quinazolinedione rings is 74.1 (1)°, giving a twisted conformation for the overall molecule. The crystal structure is dominated by common intermolecular *R*_2_^2^(8) hydrogen-bonded ring motifs formed through N3—H···O2^i^ hydrogen bonds (Table 2). These centrosymmetric dimers are extended through weak C—H···O hydrogen-bonding associations involving the second carbonyl group in a bifurcated *R*_1_^2^(7) motif (C18—H···O10^ii^, C25—H···O10^ii^), giving an expanded cyclic *R*_2_^2^(20) motif in one-dimensional chains extending along *a* (Fig. 2).

### S2. Experimental

2-Amino-*N*-[3-(4-phenhylpiperazin-1-yl)propyl]benzamide (1.7 g, 5 mmol) was stirred in CH_2_Cl_2_ (50 ml) at room temperature and triphosgene (0.5 g, 1.7 mmol) in CH_2_Cl_2_ (10 ml) was added. The mixture was refluxed for 2 h. The organic phase was washed with water and dried over MgSO_4_. The solvent was removed under reduced pressure, to give a solid product, which was recrystallized from ethanol, affording pure pelanserin. *M*.p. 190–192 °C, yield 88%; IR (KBr): 3358 (NH), 2982 (CH), 1737 cm^-1^ (C═O). ^1^H-NMR (200 MHz, CDCl_3_, p.p.m.): δ 10.70 (s, 1H), 8.12 (d, 1H, *J* = 8.0 Hz), 7.0 (t, 1H, *J* = 8.4 Hz), 7.25 (m, 4H), 6.87 (m, 3H), 4.19 (t, 2H, *J* = 6.9 Hz), 3.12 (t, 4H, *J* = 6.0 Hz), 2.61 (m, 6H), 1.95 (q, 2H); ^13^C-NMR (50 MHz, CDCl_3_, p.p.m.): δ 162, 152, 151, 139, 135, 129, 128, 123, 119, 116, 114, 56, 53, 49, 30, 25. EIMS (*m*/*z*): 364 [*M*^+^, 3], 175 [100]. Anal. calcd. for C_21_H_24_N_4_O_2_: C 69.21, H 6.64%; found: C 69.23, H 6.58%.

### S3. Refinement

Crystals were thin plates (0.1 mm) and as a consequence, only poorly diffracting samples were obtained, hence room-temperature collected data had resolution limited to sin(θ)/λ = 0.59 Å^-1^, with 97.5% completeness. All H atoms bonded to C atoms were placed in idealized positions and refined as riding on their carrier atoms, with bond lengths fixed to 0.93 Å (aromatic CH) or 0.97 Å (methylene CH_2_). The amine H atom (H3) was found in a difference map and refined freely. For all H atoms, isotropic displacement parameters were calculated as *U*_iso_(H) = 1.2*U*_eq_(carrier atom).

### Crystal data

**Table:**

C_21_H_24_N_4_O_2_	*D*_x_ = 1.283 Mg m^−^^3^
*M**_r_* = 364.44	Melting point = 463–465 K
Monoclinic, *P*2_1_/*c*	Mo *K*α radiation, λ = 0.71073 Å
*a* = 15.7531 (17) Å	Cell parameters from 33 reflections
*b* = 5.4345 (10) Å	θ = 4.7–10.7°
*c* = 22.756 (3) Å	µ = 0.09 mm^−^^1^
β = 104.506 (9)°	*T* = 296 K
*V* = 1886.0 (5) Å^3^	Plate, yellow
*Z* = 4	0.60 × 0.30 × 0.10 mm
*F*(000) = 776	

### Data collection

**Table:**

Bruker P4 diffractometer	*R*_int_ = 0.077
Radiation source: fine-focus sealed tube	θ_max_ = 25.0°, θ_min_ = 2.5°
Graphite monochromator	*h* = 0→18
2θ/ω scans	*k* = 0→6
3452 measured reflections	*l* = −27→26
3323 independent reflections	3 standard reflections every 97 reflections
1301 reflections with *I* > 2σ(*I*)	intensity decay: 1%

### Refinement

**Table:**

Refinement on *F*^2^	Primary atom site location: structure-invariant direct methods
Least-squares matrix: full	Secondary atom site location: difference Fourier map
*R*[*F*^2^ > 2σ(*F*^2^)] = 0.072	Hydrogen site location: mixed
*wR*(*F*^2^) = 0.148	H atoms treated by a mixture of independent and constrained refinement
*S* = 0.99	*w* = 1/[σ^2^(*F*_o_^2^) + (0.0399*P*)^2^] where *P* = (*F*_o_^2^ + 2*F*_c_^2^)/3
3323 reflections	(Δ/σ)_max_ < 0.001
247 parameters	Δρ_max_ = 0.18 e Å^−^^3^
0 restraints	Δρ_min_ = −0.19 e Å^−^^3^
0 constraints	

### Fractional atomic coordinates and isotropic or equivalent isotropic displacement parameters (Å^2^)

**Table:**

	*x*	*y*	*z*	*U*_iso_*/*U*_eq_	
N1	0.8261 (2)	0.4446 (7)	0.56297 (15)	0.0441 (10)	
C2	0.8955 (3)	0.4694 (10)	0.5359 (2)	0.0476 (13)	
O2	0.90428 (19)	0.3299 (6)	0.49494 (13)	0.0615 (10)	
N3	0.9524 (2)	0.6547 (8)	0.55692 (17)	0.0510 (11)	
H3	1.004 (3)	0.669 (8)	0.5430 (17)	0.061*	
C4	0.9477 (3)	0.8081 (9)	0.60436 (19)	0.0437 (12)	
C5	1.0100 (3)	0.9929 (9)	0.6239 (2)	0.0530 (14)	
H5A	1.0556	1.0136	0.6051	0.064*	
C6	1.0035 (3)	1.1443 (10)	0.6712 (2)	0.0602 (14)	
H6A	1.0447	1.2681	0.6842	0.072*	
C7	0.9361 (3)	1.1131 (10)	0.6995 (2)	0.0592 (14)	
H7A	0.9326	1.2143	0.7318	0.071*	
C8	0.8741 (3)	0.9326 (9)	0.6800 (2)	0.0536 (14)	
H8A	0.8282	0.9145	0.6986	0.064*	
C9	0.8798 (3)	0.7762 (9)	0.63228 (18)	0.0416 (12)	
C10	0.8130 (3)	0.5898 (9)	0.61014 (19)	0.0457 (12)	
O10	0.7479 (2)	0.5615 (6)	0.62947 (13)	0.0649 (10)	
C11	0.7659 (3)	0.2377 (9)	0.54229 (18)	0.0488 (13)	
H11A	0.7447	0.1788	0.5763	0.059*	
H11B	0.7982	0.1045	0.5296	0.059*	
C12	0.6878 (3)	0.3001 (9)	0.49053 (18)	0.0506 (13)	
H12A	0.7075	0.3740	0.4575	0.061*	
H12B	0.6502	0.4169	0.5041	0.061*	
C13	0.6375 (3)	0.0642 (9)	0.46908 (18)	0.0502 (13)	
H13A	0.6737	−0.0416	0.4510	0.060*	
H13B	0.6271	−0.0211	0.5041	0.060*	
N14	0.5535 (2)	0.1028 (7)	0.42500 (15)	0.0435 (10)	
C15	0.5664 (3)	0.1797 (9)	0.36668 (18)	0.0551 (14)	
H15A	0.6014	0.0573	0.3524	0.066*	
H15B	0.5985	0.3337	0.3717	0.066*	
C16	0.4801 (3)	0.2128 (9)	0.31949 (19)	0.0605 (14)	
H16A	0.4476	0.3471	0.3317	0.073*	
H16B	0.4917	0.2565	0.2809	0.073*	
N17	0.4269 (2)	−0.0093 (7)	0.31190 (15)	0.0432 (10)	
C18	0.4153 (3)	−0.0908 (9)	0.37070 (18)	0.0528 (13)	
H18A	0.3833	−0.2451	0.3657	0.063*	
H18B	0.3811	0.0302	0.3862	0.063*	
C19	0.5027 (3)	−0.1247 (9)	0.41534 (19)	0.0555 (14)	
H19A	0.4935	−0.1805	0.4538	0.067*	
H19B	0.5356	−0.2507	0.4004	0.067*	
C20	0.3524 (3)	−0.0201 (9)	0.26258 (19)	0.0446 (12)	
C21	0.3356 (3)	0.1559 (10)	0.2177 (2)	0.0651 (16)	
H21A	0.3723	0.2919	0.2209	0.078*	
C22	0.2645 (3)	0.1331 (11)	0.1677 (2)	0.0708 (17)	
H22A	0.2545	0.2541	0.1378	0.085*	
C23	0.2089 (3)	−0.0630 (11)	0.1614 (2)	0.0643 (15)	
H23A	0.1614	−0.0777	0.1278	0.077*	
C24	0.2254 (3)	−0.2380 (11)	0.2063 (2)	0.0656 (15)	
H24A	0.1878	−0.3722	0.2031	0.079*	
C25	0.2961 (3)	−0.2202 (10)	0.2562 (2)	0.0559 (14)	
H25A	0.3061	−0.3428	0.2857	0.067*	

### Atomic displacement parameters (Å^2^)

**Table:**

	*U*^11^	*U*^22^	*U*^33^	*U*^12^	*U*^13^	*U*^23^
N1	0.033 (2)	0.057 (3)	0.043 (2)	−0.005 (2)	0.0113 (18)	0.003 (2)
C2	0.032 (3)	0.069 (4)	0.038 (3)	0.001 (3)	0.000 (2)	0.000 (3)
O2	0.059 (2)	0.079 (3)	0.0492 (19)	−0.012 (2)	0.0193 (17)	−0.019 (2)
N3	0.037 (2)	0.065 (3)	0.052 (2)	−0.010 (2)	0.013 (2)	−0.008 (2)
C4	0.039 (3)	0.049 (3)	0.040 (3)	0.002 (3)	0.005 (2)	0.001 (3)
C5	0.040 (3)	0.063 (4)	0.053 (3)	0.001 (3)	0.005 (2)	0.012 (3)
C6	0.057 (3)	0.056 (4)	0.061 (3)	−0.002 (3)	0.002 (3)	−0.002 (3)
C7	0.065 (3)	0.056 (4)	0.054 (3)	0.006 (3)	0.010 (3)	−0.005 (3)
C8	0.047 (3)	0.063 (4)	0.050 (3)	0.014 (3)	0.011 (2)	0.007 (3)
C9	0.039 (3)	0.048 (3)	0.034 (2)	0.012 (3)	0.003 (2)	0.007 (2)
C10	0.042 (3)	0.051 (3)	0.042 (3)	0.006 (3)	0.007 (2)	0.004 (3)
O10	0.054 (2)	0.079 (3)	0.073 (2)	−0.009 (2)	0.0345 (18)	−0.008 (2)
C11	0.036 (3)	0.061 (3)	0.049 (3)	−0.001 (3)	0.010 (2)	0.004 (3)
C12	0.044 (3)	0.052 (3)	0.048 (3)	−0.005 (3)	−0.002 (2)	0.002 (3)
C13	0.048 (3)	0.051 (3)	0.048 (3)	−0.001 (3)	0.005 (2)	−0.003 (3)
N14	0.044 (2)	0.046 (3)	0.039 (2)	−0.006 (2)	0.0074 (18)	−0.005 (2)
C15	0.051 (3)	0.064 (4)	0.048 (3)	−0.015 (3)	0.008 (2)	0.000 (3)
C16	0.059 (3)	0.064 (4)	0.055 (3)	−0.016 (3)	0.007 (3)	0.006 (3)
N17	0.045 (2)	0.048 (3)	0.038 (2)	−0.010 (2)	0.0131 (18)	0.000 (2)
C18	0.047 (3)	0.062 (4)	0.048 (3)	−0.018 (3)	0.008 (2)	−0.001 (3)
C19	0.060 (3)	0.060 (4)	0.045 (3)	−0.011 (3)	0.012 (2)	0.000 (3)
C20	0.044 (3)	0.050 (3)	0.039 (3)	0.002 (3)	0.008 (2)	−0.010 (3)
C21	0.077 (4)	0.067 (4)	0.044 (3)	−0.011 (3)	0.000 (3)	−0.002 (3)
C22	0.076 (4)	0.076 (4)	0.051 (3)	0.001 (4)	0.000 (3)	0.008 (3)
C23	0.049 (3)	0.087 (4)	0.054 (3)	0.000 (4)	0.007 (3)	−0.011 (3)
C24	0.053 (3)	0.079 (4)	0.064 (3)	−0.022 (3)	0.015 (3)	−0.016 (4)
C25	0.049 (3)	0.058 (4)	0.056 (3)	−0.005 (3)	0.004 (3)	−0.006 (3)

### Geometric parameters (Å, º)

**Table:**

N1—C2	1.389 (5)	C13—H13B	0.9700
N1—C10	1.389 (5)	N14—C15	1.453 (5)
N1—C11	1.470 (5)	N14—C19	1.459 (5)
C2—O2	1.235 (5)	C15—C16	1.518 (5)
C2—N3	1.353 (5)	C15—H15A	0.9700
N3—C4	1.381 (5)	C15—H15B	0.9700
N3—H3	0.95 (4)	C16—N17	1.454 (5)
C4—C9	1.385 (5)	C16—H16A	0.9700
C4—C5	1.397 (6)	C16—H16B	0.9700
C5—C6	1.379 (6)	N17—C20	1.407 (5)
C5—H5A	0.9300	N17—C18	1.464 (5)
C6—C7	1.384 (6)	C18—C19	1.504 (5)
C6—H6A	0.9300	C18—H18A	0.9700
C7—C8	1.377 (6)	C18—H18B	0.9700
C7—H7A	0.9300	C19—H19A	0.9700
C8—C9	1.400 (6)	C19—H19B	0.9700
C8—H8A	0.9300	C20—C21	1.375 (6)
C9—C10	1.456 (6)	C20—C25	1.388 (6)
C10—O10	1.223 (5)	C21—C22	1.388 (6)
C11—C12	1.513 (5)	C21—H21A	0.9300
C11—H11A	0.9700	C22—C23	1.364 (6)
C11—H11B	0.9700	C22—H22A	0.9300
C12—C13	1.522 (6)	C23—C24	1.373 (6)
C12—H12A	0.9700	C23—H23A	0.9300
C12—H12B	0.9700	C24—C25	1.381 (5)
C13—N14	1.462 (5)	C24—H24A	0.9300
C13—H13A	0.9700	C25—H25A	0.9300
			
C2—N1—C10	125.0 (4)	C15—N14—C19	107.6 (3)
C2—N1—C11	116.7 (4)	C15—N14—C13	111.0 (3)
C10—N1—C11	118.2 (4)	C19—N14—C13	110.5 (4)
O2—C2—N3	122.3 (4)	N14—C15—C16	112.0 (4)
O2—C2—N1	121.6 (5)	N14—C15—H15A	109.2
N3—C2—N1	116.1 (5)	C16—C15—H15A	109.2
C2—N3—C4	124.4 (4)	N14—C15—H15B	109.2
C2—N3—H3	119 (3)	C16—C15—H15B	109.2
C4—N3—H3	115 (3)	H15A—C15—H15B	107.9
N3—C4—C9	118.8 (5)	N17—C16—C15	111.9 (4)
N3—C4—C5	120.7 (5)	N17—C16—H16A	109.2
C9—C4—C5	120.4 (5)	C15—C16—H16A	109.2
C6—C5—C4	119.6 (5)	N17—C16—H16B	109.2
C6—C5—H5A	120.2	C15—C16—H16B	109.2
C4—C5—H5A	120.2	H16A—C16—H16B	107.9
C5—C6—C7	120.4 (5)	C20—N17—C16	118.0 (4)
C5—C6—H6A	119.8	C20—N17—C18	116.5 (3)
C7—C6—H6A	119.8	C16—N17—C18	110.1 (3)
C8—C7—C6	120.1 (5)	N17—C18—C19	110.6 (3)
C8—C7—H7A	119.9	N17—C18—H18A	109.5
C6—C7—H7A	119.9	C19—C18—H18A	109.5
C7—C8—C9	120.3 (5)	N17—C18—H18B	109.5
C7—C8—H8A	119.8	C19—C18—H18B	109.5
C9—C8—H8A	119.8	H18A—C18—H18B	108.1
C4—C9—C8	119.1 (5)	N14—C19—C18	111.9 (4)
C4—C9—C10	120.1 (4)	N14—C19—H19A	109.2
C8—C9—C10	120.6 (5)	C18—C19—H19A	109.2
O10—C10—N1	120.5 (5)	N14—C19—H19B	109.2
O10—C10—C9	124.1 (5)	C18—C19—H19B	109.2
N1—C10—C9	115.4 (4)	H19A—C19—H19B	107.9
N1—C11—C12	114.3 (4)	C21—C20—C25	117.9 (4)
N1—C11—H11A	108.7	C21—C20—N17	122.0 (5)
C12—C11—H11A	108.7	C25—C20—N17	119.9 (4)
N1—C11—H11B	108.7	C20—C21—C22	120.8 (5)
C12—C11—H11B	108.7	C20—C21—H21A	119.6
H11A—C11—H11B	107.6	C22—C21—H21A	119.6
C11—C12—C13	108.5 (4)	C23—C22—C21	121.4 (5)
C11—C12—H12A	110.0	C23—C22—H22A	119.3
C13—C12—H12A	110.0	C21—C22—H22A	119.3
C11—C12—H12B	110.0	C22—C23—C24	117.8 (5)
C13—C12—H12B	110.0	C22—C23—H23A	121.1
H12A—C12—H12B	108.4	C24—C23—H23A	121.1
N14—C13—C12	114.1 (4)	C23—C24—C25	121.8 (5)
N14—C13—H13A	108.7	C23—C24—H24A	119.1
C12—C13—H13A	108.7	C25—C24—H24A	119.1
N14—C13—H13B	108.7	C24—C25—C20	120.2 (5)
C12—C13—H13B	108.7	C24—C25—H25A	119.9
H13A—C13—H13B	107.6	C20—C25—H25A	119.9
			
C10—N1—C2—O2	178.3 (4)	C10—N1—C11—C12	92.2 (4)
C11—N1—C2—O2	2.5 (6)	N1—C11—C12—C13	173.4 (4)
C10—N1—C2—N3	−1.7 (6)	C11—C12—C13—N14	171.7 (3)
C11—N1—C2—N3	−177.5 (4)	C12—C13—N14—C15	71.1 (5)
O2—C2—N3—C4	−176.9 (4)	C12—C13—N14—C19	−169.6 (4)
N1—C2—N3—C4	3.1 (6)	C19—N14—C15—C16	57.1 (5)
C2—N3—C4—C9	−1.2 (6)	C13—N14—C15—C16	178.1 (4)
C2—N3—C4—C5	179.0 (4)	N14—C15—C16—N17	−56.3 (5)
N3—C4—C5—C6	179.7 (4)	C15—C16—N17—C20	−169.2 (4)
C9—C4—C5—C6	−0.1 (6)	C15—C16—N17—C18	53.8 (5)
C4—C5—C6—C7	0.4 (7)	C20—N17—C18—C19	167.0 (4)
C5—C6—C7—C8	−1.0 (7)	C16—N17—C18—C19	−55.2 (5)
C6—C7—C8—C9	1.3 (7)	C15—N14—C19—C18	−59.3 (5)
N3—C4—C9—C8	−179.4 (4)	C13—N14—C19—C18	179.4 (3)
C5—C4—C9—C8	0.5 (6)	N17—C18—C19—N14	59.6 (5)
N3—C4—C9—C10	−2.3 (6)	C16—N17—C20—C21	8.9 (6)
C5—C4—C9—C10	177.5 (4)	C18—N17—C20—C21	143.2 (4)
C7—C8—C9—C4	−1.1 (6)	C16—N17—C20—C25	−175.1 (4)
C7—C8—C9—C10	−178.1 (4)	C18—N17—C20—C25	−40.7 (6)
C2—N1—C10—O10	176.8 (4)	C25—C20—C21—C22	−0.1 (7)
C11—N1—C10—O10	−7.4 (6)	N17—C20—C21—C22	176.1 (4)
C2—N1—C10—C9	−1.5 (6)	C20—C21—C22—C23	0.3 (8)
C11—N1—C10—C9	174.3 (3)	C21—C22—C23—C24	0.1 (8)
C4—C9—C10—O10	−174.7 (4)	C22—C23—C24—C25	−0.7 (8)
C8—C9—C10—O10	2.3 (7)	C23—C24—C25—C20	0.9 (7)
C4—C9—C10—N1	3.5 (6)	C21—C20—C25—C24	−0.5 (7)
C8—C9—C10—N1	−179.5 (4)	N17—C20—C25—C24	−176.7 (4)
C2—N1—C11—C12	−91.7 (4)		

### Hydrogen-bond geometry (Å, º)

**Table:**

*D*—H···*A*	*D*—H	H···*A*	*D*···*A*	*D*—H···*A*
N3—H3···O2^i^	0.95 (4)	1.85 (4)	2.799 (5)	171 (4)
C18—H18*A*···O10^ii^	0.97	2.71	3.625 (6)	157
C25—H25*A*···O10^ii^	0.93	2.59	3.404 (6)	147

Symmetry codes: (i) −*x*+2, −*y*+1, −*z*+1; (ii) −*x*+1, −*y*, −*z*+1.
